# Angiotensin receptor-neprilysin inhibitor therapy and recurrence of atrial fibrillation after radiofrequency catheter ablation: A propensity-matched cohort study

**DOI:** 10.3389/fcvm.2022.932780

**Published:** 2022-08-04

**Authors:** Youzheng Dong, Shucai Xiao, Jinwu He, Kaixin Shi, Si Chen, Deping Liu, Bin Huang, Zhenyu Zhai, Juxiang Li

**Affiliations:** Department of Cardiovascular Medicine, The Second Affiliated Hospital of Nanchang University, Nanchang, China

**Keywords:** ARNI, atrial fibrillation, RFCA, follow-up, recurrence

## Abstract

**Background:**

Compared with conventional medicines, angiotensin receptor-neprilysin inhibitor (ARNI) could further improve the prognosis for multiple cardiovascular diseases, such as heart failure, hypertension, and myocardial infarction. However, the relationship between ARNI therapy and the recurrence of atrial fibrillation (AF) after radiofrequency catheter ablation is currently unknown.

**Methods:**

This study is a retrospective cohort study. Patients with consecutive persistent or paroxysmal AF undergoing first-time radiofrequency ablation were enrolled from February 2018 to October 2021. We compared the risk of AF recurrence in patients with catheter ablation who received ARNI with the risk of AF recurrence in those who received the angiotensin-converting enzyme inhibitor (ACEI). The propensity-score matched analysis was conducted to examine the effectiveness of ARNI. We used a Cox regression model to evaluate AF recurrence events.

**Results:**

Among 679 eligible patients, 155 patients with ARNI treatment and 155 patients with ACEI treatment were included in the analyses. At a median follow-up of 228 (196–322) days, ARNI as compared with ACEI was associated with a lower risk of AF recurrence [adjusted hazard ratio (HR), 0.39; 95% confidence interval (CI), 0.24–0.63; *p* < 0.001]. In addition, no interaction was found in the subgroup analysis.

**Conclusion:**

Angiotensin receptor-neprilysin inhibitor treatment was associated with a decreased risk of AF recurrence after first-time radiofrequency catheter ablation.

## Introduction

According to Global Burden of Disease estimates, more than 30 million individuals have been affected by atrial fibrillation (AF) and associated disorders (1). AF often results in serious complications, such as death, stroke, left ventricle dysfunction or heart failure (HF), cognitive decline, depression, and impaired quality of life (2–6). The incidence of AF has increased over the past 20 years and is expected to increase further due to longevity, multi-comorbidity, and more accessible primary healthcare (7). Radiofrequency catheter ablation (RFCA) is a well-established therapeutic strategy for the treatment of AF, its primary clinical benefit is a decrease in arrhythmia-related symptoms (8, 9). Although RFCA is superior to antiarrhythmic drugs (AADs) for rhythm control, many patients require reoperation, and the risk of long-term recurrence after ablation remains high (10, 11).

Angiotensin receptor-neprilysin inhibitor (ARNI), a kind of compound preparation, is becoming increasingly important in the field of clinical therapeutics for cardiovascular diseases. It exhibits a distinctive dual mechanism of action, simultaneously suppressing the enkephalinase and angiotensin II (Ang II) receptor, thereby increasing the levels of natriuretic peptides (NPs) and decreasing the levels of Ang II (12). NPs with high levels have beneficial effects on the cardiovascular system, leading to natriuresis/diuresis, vasodilation, and reducing fibrosis and hypertrophy, thereby further antagonizing the adverse effects of the sympathetic nervous system (SNS) excitation and the renin-angiotensin-aldosterone system (RAAS) activation (12). According to the latest guidelines for the management of HF, ARNI is recommended as the preferred agent for patients with the New York Heart Association (NYHA) class II–III, if patients are unable to tolerate ARNI, the patient may be switched to angiotensin-converting enzyme inhibitor (ACEI) (13). For the treatment of hypertension, a pooled analysis demonstrated that ARNI (200 mg/day) is associated with a 4.94 mmHg decrease in systolic blood pressure (SBP) and a 3.69 mmHg decrease in 24-h ambulatory SBP vs. the ARB (14). Additionally, the results of some small sample studies showed that ARNI appears to further improve short-term clinical outcomes in patients with acute myocardial infarction (MI) (15–18).

Given the vasodilatory, anti-cardiac remodeling, and sympatholytic effects of ARNI, along with the electrical isolation with RFCA, the use of ARNI in patients with ablation appears to be an attractive option for preventing, reducing, and delaying the recurrence of atrial arrhythmias. Therefore, in the context of contemporary practices, we conducted a study to evaluate the association between ARNI therapy and the recurrence of AF after first-time RFCA in patients with AF.

## Materials and methods

### Study design and population

This study was a retrospective cohort study. We consecutively enrolled patients with non-valvular, drug-refractory AF who underwent RFCA at the Second Affiliated Hospital of Nanchang University from February 2018 to October 2021. The exclusion criteria were as follows: (1) patients who did not receive ACEI or ARNI, or the duration shorter than 3 months; (2) previous history of ablation; (3) pre-excited AF; and (4) implanted pacemaker or defibrillator. This study has been conducted in accordance with the Declaration of Helsinki (2013) and has been approved by the ethics committee at the Second Affiliated Hospital of Nanchang University.

### Radiofrequency ablation strategy

Oral anticoagulant was given to all subjects at least 1 month before RFCA. Two-dimensional echocardiography, enhanced cardiac computed tomography, 24-h Holter-electrocardiogram (ECG), and transesophageal echocardiography (TEE) were routinely performed for all patients. AADs were discontinued for ≥5 half-lives prior to RFCA.

A decapolar catheter, advanced through the coronary sinus, was introduced *via* the left femoral vein percutaneously for stimulation and electroanatomical reference. After establishing LA access, heparin was administered, and the activated clotting time was maintained at 300–350 s. A 20-polar circular-shaped catheter (Lasso, Biosense Webster, Diamond Bar, CA, United States) was placed in the pulmonary veins (PVs) and a 3.5-mm irrigated-tip ablation catheter (NAVISTAR THERMOCOOL, Biosense Webster, Diamond Bar, CA, United States) was introduced by the right femoral vein. A 3D map was conducted by using the CARTO^®^ system (CARTO 3, Biosense Webster, Diamond Bar, CA, United States). The procedure consisted of the following stepwise strategy: a pulmonary vein antrum isolation (PV), more than 5 mm from the PV Ostia, was performed to achieve atrial and PV bidirectional electrical conduction block with a maximum power of 30–40 W, a maximal temperature of 43°C, and an irrigation rate of 30 ml/min. The endpoint of pulmonary vein isolation (PVI) was a complete bidirectional conduction block between the left atrium and PV. After PVI, if a non-PV trigger was present, additional isolation was performed to eliminate the non-PV trigger. After trigger ablation, in patients without AF termination and paroxysmal AF patients with inducibility of AF lasting >5 min, additional ablation, such as LA linear (LA roofline and mitral isthmus line), cavotricuspid isthmus, and complex fractionated atrial electrogram (CFAE) ablation was performed at the operator’s discretion. If the patient remained in AF after the above ablation, external electrical cardioversion was given immediately. Oral anticoagulation was continued after the RFCA and AADs for 3 months.

### Angiotensin-converting enzyme inhibitor or angiotensin receptor-neprilysin inhibitor therapy

After RFCA, the selection of ACEI or ARNI was determined based on the corresponding guidelines and physicians’ decisions (19, 20), regardless of whether or not they had received prior ACEI/ARB or ARNI. If patients were switched to ARNI after RFCA immediately, washout periods of at least 36 h were established for patients previously treated with ACEI or ARB.

### Patient follow-up and study definitions

Patients were visited 14–21 days after RFCA and every 1–3 months thereafter. ECG and 24-h Holter ECG were performed at each follow-up visit. If patients felt palpitations, dyspnea, fatigue, chest tightness/pain, poor effort tolerance, dizziness, syncope, etc., they were asked to immediately visit the hospital. After 3 months, AADs were weaned, and oral anticoagulation was continued subsequently depending on CHA2DS2-VASc thereafter. The primary endpoint was defined as the presence of at least one episode of AF, atrial flutter, or atrial tachycardia >30 s in any 12-lead ECG or 24 h Holter- monitoring after a 3-month blanking period. If the patients discontinued the ARNI or ACEI during follow-up due to intolerance or other reasons, their data were censored. The duration of AF was defined as the time interval between the initial onset of AF-related symptoms and the last diagnosis of AF. Renal insufficiency was defined as serum creatinine >133 μmol/L (1.5 mg/dl).

### Statistical analysis

Numeric variables were summarized as means (standard deviation, SD) or median (inter-quartile range, IQR) and analyzed by Student’s *t*-test or the Wilcoxon rank-sum test. Categorical variables were summarized as frequencies (percentages) and analyzed by the χ^2^ test or Fisher’s exact test. We conducted a propensity score-matched analysis using all variables listed in Table 1 to establish a matched cohort between patients with ACEI and patients with ARNI. Propensity score matching (PSM) was conducted using the nearest neighbor matching without replacement, using a 1:1 matching protocol with a caliper width of 0.2. The balance of matched cohorts was evaluated with standardized differences, and absolute values >20% were considered unacceptably imbalanced (21). All subsequent analyses were based on the propensity score–matched cohorts. The Kaplan–Meier method with a log-rank test was used to assess the cumulative risks of AF recurrence as predefined after a blanking period of 3 months during the follow-up period. The multivariate Cox proportional hazard regression analyses were conducted to compare the risks of AF recurrence between ACEI and ARNI, and estimate the adjusted hazard ratios (HRs) with 95% confidence intervals (95% CIs), a crude model was unadjusted, model I was adjusted for age, sex, body mass index (BMI), duration of AF, left atrial diameter (LAD), and AF type, and model II was adjusted for age, sex, BMI, duration of AF, LAD, AF type, smoking status, alcohol drinking status, estimated glomerular filtration rate (eGFR), serum creatinine, HbA1c, total cholesterol (TC), triglyceride (TG), low-density lipoprotein cholesterol (LDL-c), additional ablation, beta-blockers, calcium channel blocker (CCB), statins, mineralocorticoid receptor antagonist (MRA), diuretics, digoxin, AADs, and history of disease [hypertension, diabetes mellitus, coronary heart disease (CAD), HF, and renal insufficiency]. Schoenfeld residuals were used to validate the Cox proportional hazards regression assumptions. In addition, a subgroup treatment effect forest plot was generated to explore the effect of ARNI on the recurrence of AF, with the adjusted HRs and 95% CIs. Clinical subgroups included age, sex, AF type, duration of AF, hypertension, diabetes, CAD, left ventricular ejection fraction (LVEF), and additional ablation. In subgroup analysis, variables included for adjustment were BMI, smoking status, alcohol drinking status, eGFR, HbA1c, TC, TG, LDL-c, beta-blockers, CCB, statins, MRA, diuretics, digoxin, and AADs. Tests of interaction were conducted to evaluate the heterogeneity of treatment effect across these subgroups. The value of *p* < 0.05 (two-sided) was considered statistically significant. All analyses were performed using RStudio version 1.1.414 (Boston, MA, United States) and Empower^1^ (X&Y Solutions, Inc., Boston, MA, United States).

**TABLE 1:** Baseline characteristics of the study population.

Characteristic	Before matching			After matching		
	ACEI	ARNI	*p*-Value	ACEI	ARNI	*p*-Value
*N*	470	209		155	155	
Age (years)	64.34 (10.96)	65.30 (10.08)	0.278	65.82 (9.63)	65.03 (10.03)	0.481
Female (%)	218 (46.38%)	80 (38.28%)	0.049	66 (42.58%)	60 (38.71%)	0.488
BMI (kg/m^2^)	23.91 (3.37)	24.43 (3.19)	0.055	24.85 (3.72)	24.42 (3.18)	0.241
AF type (%)			<0.001			0.230
Paroxysmal	431 (91.70%)	158 (75.60%)		136 (87.74%)	129 (83.23%)	
Persistent	39 (8.30%)	51 (24.40%)		19 (12.26%)	26 (16.77%)	
Duration of AF (months)	12.00 (2.00–48.00)	12.00 (2.00–38.00)	0.211	12.00 (2.00–36.00)	12.00 (3.00–45.00)	0.394
**Medical history**						
Hypertension (%)	345 (73.40%)	116 (55.50%)	<0.001	113 (72.90%)	104 (67.10%)	0.265
Diabetes mellitus (%)	97 (20.64%)	40 (19.14%)	0.653	32 (20.65%)	29 (18.71%)	0.668
Coronary artery disease (%)	85 (18.09%)	48 (22.97%)	0.139	35 (22.58%)	36 (23.23%)	0.892
Heart failure (%)	285 (60.64%)	168 (80.38%)	<0.001	129 (83.23%)	130 (83.87%)	0.878
Renal insufficiency (%)	33 (7.02%)	43 (20.57%)	<0.001	26 (16.77%)	24 (15.48%)	0.757
Current smoking (%)	89 (18.94%)	43 (20.57%)	0.619	30 (19.35%)	35 (22.58%)	0.485
Current alcohol (%)	58 (12.34%)	30 (14.35%)	0.471	23 (14.84%)	25 (16.13%)	0.754
HbA1c (%)	5.80 (0.80)	5.77 (0.56)	0.616	5.83 (0.60)	5.78 (0.55)	0.730
TC (mmol/L)	4.34 (1.02)	4.17 (0.99)	0.046	4.28 (1.02)	4.21 (0.95)	0.536
TG (mmol/L)	1.47 (0.91)	1.56 (1.30)	0.275	1.54 (1.12)	1.58 (1.24)	0.749
HDL-c (mmol/L)	1.17 ± 0.30	1.07 ± 0.30	<0.001	1.10 (0.25)	1.10 (0.30)	0.893
LDL-c (mmol/L)	2.50 (0.76)	2.37 (0.69)	0.038	2.44 (0.75)	2.43 (0.65)	0.840
eGFR (mL/min per 1.73 m^2^)	84.09 (21.15)	76.04 (26.64)	<0.001	76.96 (20.37)	78.16 (26.56)	0.655
Serum creatinine (umol/L)	82.18 (43.77)	100.11 (75.38)	<0.001	92.37 (69.30)	96.14 (72.13)	0.639
BNP (pg/ml)	120.12 (45.32-289.07)	239.95 (84.07-641.58)	<0.001	178.69 (73.60–373.57)	159.46 (76.37–400.05)	0.493
LAD (mm)	36.51 (5.41)	39.37 (5.96)	<0.001	38.92 (5.14)	38.77 (5.63)	0.801
LVEF (%)	54.74 (7.92)	45.67 (12.06)	<0.001	50.28 (9.03)	48.60 (10.57)	0.133
PV isolation (%)	470 (100%)	209 (100%)	0.505	155 (100%)	155 (100%)	1.000
SVC isolation (%)	62 (13.19%)	43 (20.57%)	0.014	26 (16.77%)	28 (18.06%)	0.765
LA CFAE ablation (%)	11 (2.34%)	11 (5.26%)	0.047	6 (3.87%)	6 (3.87%)	1.000
LA linear ablation (%)	156 (33.19%)	91 (43.54%)	0.010	57 (36.77%)	64 (41.29%)	0.451
CTI ablation (%)	94 (20.00%)	49 (23.44%)	0.310	31 (20.00%)	32 (20.65%)	0.888
NYHA functional class (%)			<0.001			0.389
I	184 (39.15%)	42 (20.10%)		36 (23.23%)	37 (23.87%)	
II	255 (54.26%)	139 (66.51%)		107 (69.03%)	106 (68.39%)	
III	26 (5.53%)	17 (8.13%)		10 (6.45%)	6 (3.87%)	
IV	5 (1.06%)	11 (5.26%)		2 (1.29%)	6 (3.87%)	
CHA_2_DS_2_-VASc score	2.30 (1.59)	2.24 (1.44)	0.660	2.37 (1.55)	2.25 (1.45)	0.473
**Drugs (%)**						
Beta-blockers	376 (80.00%)	180 (86.12%)	0.056	132 (85.16%)	136 (87.74%)	0.507
CCB	141 (30.00%)	64 (30.62%)	0.871	50 (32.26%)	49 (31.61%)	0.903
Statins	196 (41.70%)	92 (44.02%)	0.573	79 (50.97%)	73 (47.10%)	0.495
MRA	51 (10.85%)	42 (20.10%)	0.001	26 (16.77%)	25 (16.13%)	0.878
Diuretics	89 (18.94%)	51 (24.40%)	0.104	39 (25.16%)	39 (25.16%)	1.000
Digoxin	41 (8.72%)	30 (14.35%)	0.027	20 (12.90%)	20 (12.90%)	1.000
Anticoagulation			0.904			0.783
Warfarin	17 (3.62%)	10 (4.78%)		10 (6.45%)	8 (5.16%)	
Dabigatran	293 (62.34%)	130 (62.20%)		95 (61.29%)	103 (66.45%)	
Rivaroxaban	145 (30.85%)	63 (30.14%)		43 (27.74%)	39 (25.16%)	
AADs			0.463			0.851
Amiodarone	329 (70.00%)	139 (66.51%)		102 (65.81%)	100 (64.52%)	
Propafenone	57 (12.13%)	21 (10.05%)		20 (12.90%)	17 (10.97%)	
Dronedarone	8 (1.70%)	6 (2.87%)		3 (1.94%)	5 (3.23%)	
Sotalol	3 (0.64%)	1 (0.48%)		2 (1.29%)	1 (0.65%)	

Data are presented as mean ± standard deviation (SD), or median (IQR) and percentages.

ACEI, angiotensin converting enzyme inhibitor; ARNI, angiotensin receptor neprilysin inhibitor; BMI, body mass index; AF, atrial fibrillation; HbA1c, hemoglobin A1c; TC, total cholesterol; TG, triglyceride; HDL-c, high density lipoprotein cholesterol; LDL-c, low density lipoprotein cholesterol; eGFR, estimated glomerular filtration rate; BNP, brain natriuretic peptide; LAD, left atrial diameter; LVEF, left ventricle ejection fraction; PV, pulmonary vein; SVC, superior vena cava; LA, left atrium; CFAE, complex fractionated atrial electrogram; CTI, cavotricuspid isthmus; NYHA, New York Heart Association; MRA, mineralocorticoid receptor antagonist; CCB, calcium channel blockers; AADs, antiarrhythmic drugs.

## Results

### Characteristics of the study population

**FIGURE 1:**
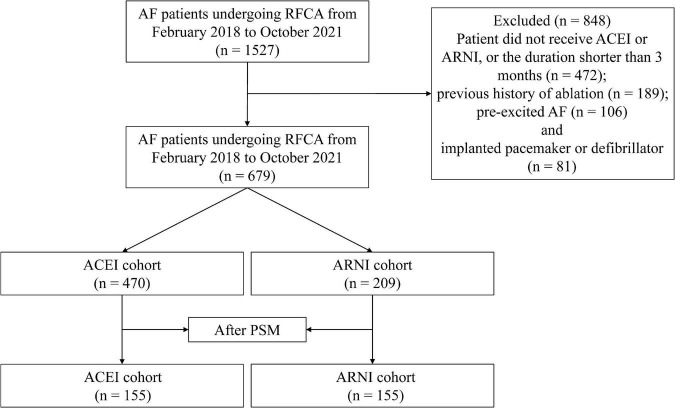
Study cohort flow diagram. AF, atrial fibrillation; RFCA, radiofrequency catheter ablation; ACEI, angiotensin-converting enzyme inhibitor; ARNI, angiotensin receptor-neprilysin inhibitor; PSM, propensity score matching.

A total of 1,527 patients who received RFCA were screened, and 679 were eligible for the analysis (Figure 1). In addition, 470 (69.22%) of these patients received ACEI, and 209 (30.78%) received ARNI following RFCA. Before PSM, there were differences between ACEI and ARNI in some baseline variables, such as sex, AF type, history of HF, history of renal insufficiency, TC, HDL-c, LDL-c, eGFR, serum creatinine, brain natriuretic peptide (BNP), LAD, LVEF, superior vena cava (SVC) isolation, LA CFAE ablation, LA linear ablation, NYHA functional class, and drugs (Table 1). After matching, no variable exhibited a large imbalance between the two cohorts. Of the 155 patients in the ARNI cohort, the mean age was 65.0 years, 37.7% were women, the mean BMI was 24.4, and 83.2% were classified as paroxysmal AF. In the ACEI cohort, the mean age was 65.8 years, 42.6% were women, the mean BMI was 24.9, and 87.7% were classified as paroxysmal AF. Additionally, 44 (28.4%) patients in the ARNI cohort and 58 (37.4%) patients in the ACEI cohort reached the target dose throughout the follow-up. In the ACEI cohort, types of drugs are shown in Table 2.

**TABLE 2:** Types of ACEIs prescribed in our study.

	*N*
Enalapril	88 (56.8%)
Benazepril	47 (30.3%)
Perindopril	20 (12.9%)

### Primary endpoint

**FIGURE 2:**
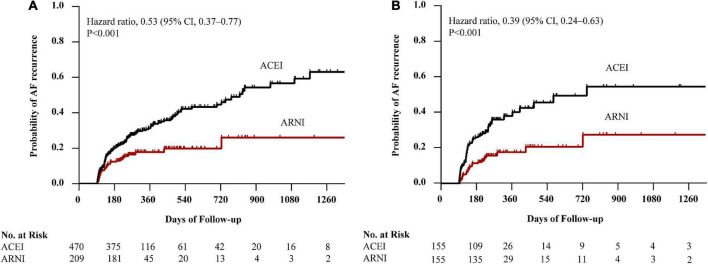
Kaplan–Meier curves of cumulative probability of AF recurrence before PSM **(A)** and after PSM **(B)**. The HR after PSM was adjusted for age, sex, BMI, duration of AF, LAD, AF type, smoking status, alcohol drinking status, eGFR, serum creatinine, HbA1c, TC, TG, LDL-c, additional ablation, beta-blockers, CCB, statins, MRA, diuretics, digoxin, AADs, and history of diseases (hypertension, diabetes mellitus, CAD, HF, and renal insufficiency). AF, atrial fibrillation; ACEI, angiotensin-converting enzyme inhibitor; ARNI, angiotensin receptor-neprilysin inhibitor; PSM, propensity score matching; HR, hazard ratio; CI, confidence interval; BMI, body mass index; LAD, left atrial diameter; eGFR, estimated glomerular filtration rate; TC, total cholesterol; TG, triglyceride; LDL-c, low-density lipoprotein cholesterol; CCB, calcium channel blocker; MRA, mineralocorticoid receptor antagonist; AADs, antiarrhythmic drugs; CAD, coronary heart disease; HF, heart failure.

**FIGURE 3:**
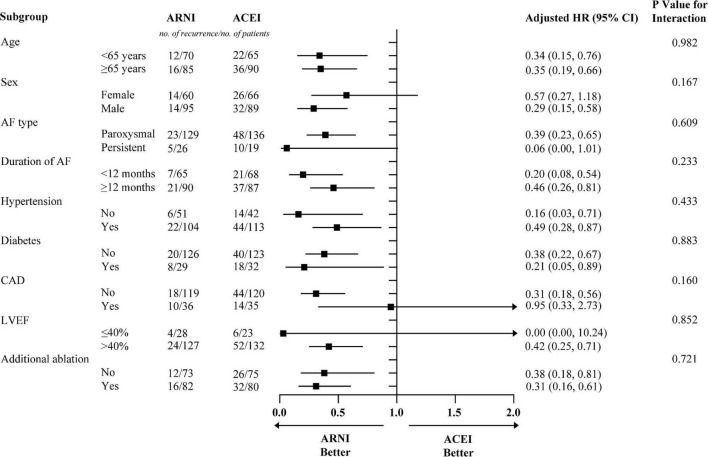
The subgroup analysis for AF recurrence rates in patients with ARNI and ACEI. Models were adjusted for BMI, smoking status, alcohol drinking status, eGFR, HbA1c, TC, TG, LDL-c, beta-blockers, CCB, statins, MRA, diuretics, digoxin, and AADs. AF, atrial fibrillation; ACEI, angiotensin-converting enzyme inhibitor; ARNI, angiotensin receptor-neprilysin inhibitor; BMI, body mass index; eGFR, estimated glomerular filtration rate; TC, total cholesterol; TG, triglyceride; LDL-c, low-density lipoprotein cholesterol; CCB, calcium channel blocker; MRA, mineralocorticoid receptor antagonist; AADs, antiarrhythmic drugs; CAD, coronary heart disease; LVEF, left ventricular ejection fraction; HR, hazard ratio; CI, confidence interval.

Before PSM, AF recurrence occurred in 152 (32.3%) patients in the ACEI cohort and 36 (17.2%) patients in the ARNI cohort after a median follow-up of 234 (IQR: 198–330) days. When compared with the ACEI, the HR for AF recurrence in the ARNI cohort was 0.53 (95% CI: 0.37–0.77; *p* < 0.001; Figure 2A). After PSM, AF recurrence occurred in 58 (37.4%) patients in the ACEI cohort and 28 (18.1%) patients in the ARNI cohort after a median follow-up of 228 (IQR: 196–322) days, and when compared with the ACEI, the crude HR and adjusted HR for AF recurrence in the ARNI cohort were 0.43 (95% CI: 0.27 to 0.68; *p* < 0.001; crude model), 0.44 (95% CI: 0.28–0.70; *p* < 0.001; model I), and 0.39 (95% CI: 0.24–0.63; *p* < 0.001; model II, Figure 2B and Table 3), respectively. Figure 3 demonstrates the associations between ARNI and AF recurrence in several clinical subgroups. Although the adjusted HRs for the AF recurrence were not consistent in the diabetes subgroups, there was no significant interaction for all subgroups.

**TABLE 3:** The risk of AF recurrence in the propensity-score–matched cohort.

			Crude		Model I		Model II	
	No. of patients with event	Event rate	HR (95% CI)	*p*	HR (95% CI)	*p*	HR (95% CI)	*p*
ACEI	58	37.4%	Ref.		Ref.		Ref.	
ARNI	28	18.1%	0.43 (0.27, 0.68)	<0.001	0.44 (0.28, 0.70)	<0.001	0.39 (0.24, 0.63)	<0.001

Model I was adjusted for age, sex, BMI, duration of AF, LAD, and AF type; Model II was adjusted for age, sex, BMI, duration of AF, LAD, AF type, smoking status, alcohol drinking status, eGFR, serum creatinine, HbA1c, total cholesterol, triglyceride, LDL-c, additional ablation, beta-blockers, CCB, statins, MRA, diuretics, digoxin, AADs, and history of diseases (hypertension, diabetes mellitus, coronary heart disease, heart failure, and renal insufficiency).

ACEI, angiotensin converting enzyme inhibitor; 95% CI, 95% confidence interval; BMI, body mass index; AF, atrial fibrillation; LAD, left atrial diameter; eGFR, estimated glomerular filtration rate; HbA1c, hemoglobin A1c; LDL-c, low-density lipoprotein cholesterol; CCB, calcium channel blockers; MRA, mineralocorticoid receptor antagonist; AADs, antiarrhythmic drugs.

## Discussion

Our study is the first to investigate the effect of ARNI vs. ACEI on AF recurrence in patients with AF after their first RFCA in the real world. We found that compared with ACEI, ARNI could significantly reduce the risk of AF recurrence in patients with AF following RFCA after a 3-month blanking period.

To date, effective management of AF remains complex and challenging. Numerous measures have been presented to reduce the recurrence rate of AF, such as reinforcing the identification and management of risk factors, improving ablation strategy, developing new ablation modalities, and optimizing medications. However, since the etiologic mechanisms of AF are still not fully understood, the prognosis of patients with AF has not been well improved.

**FIGURE 4:**
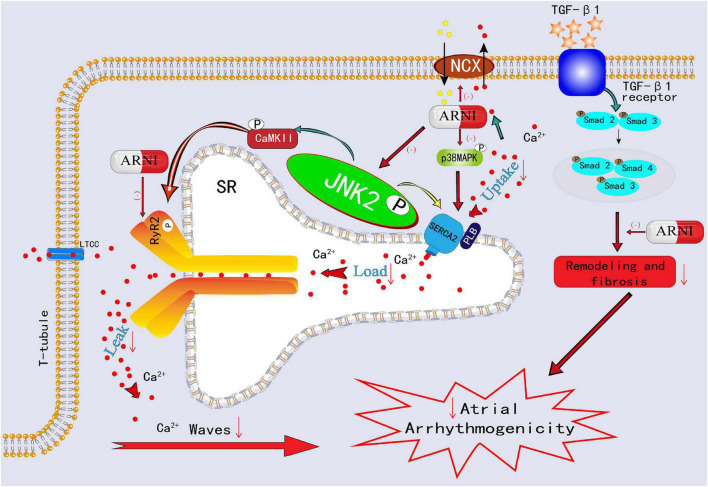
Schematic showing ARNI decreases atrial arrhythmogenicity by reversing the remodeling of RyR2 channels and NCX1 channels and inhibiting p-Smad2/3, p-JNK, and p-p38 signaling pathways.

To change the current poor situation of AF management, increasing attention is dedicated to the dissection of the molecular and electrical mechanisms of AF occurrence. Currently, electropathology, which is defined as the impairment of electrical activation (electrical and calcium remodeling) caused by molecular defects in cardiomyocytes that lead to structural damage (cardiac remodeling), is considered to play a critical role in driving AF (22, 23). Moreover, abnormal autonomic innervation (autonomic nerve remodeling) is involved in electropathology, causing markedly heterogeneous changes in atrial electrophysiology and inducing atrial tachyarrhythmia (24). Previous studies have shown that early after-depolarization (EAD) and delayed after-depolarization (DAD) may underlie PV ectopic activity (25). Although the electrical isolation of PV is the mainstream therapy for AF, AF recurrence develops in up to 70% of patients with persistent AF (PeAF) within 12 months of the first PVI (26). The primary underlying mechanisms of this ectopic activity involve electrical and calcium remodeling. During systole, a small amount of extracellular Ca^2+^ enters the cytoplasm through L-type Ca^2+^ channels (LTCC) and mediates the opening of leaky ryanodine receptor 2 (RyR2) channels, allowing Ca^2+^ in the sarcoplasmic reticulum (SR) to rapidly and abundantly enter the cytoplasm from the SR, causing myocyte contraction. During diastole, 30% of the cytosolic Ca^2+^ is mainly excluded from the cytoplasm by the sodium-calcium exchanger (NCX) and plasma membrane calcium ATPase (PMCA), and 70% of the cytosolic Ca^2+^ is pumped back to the SR by sarco-endoplasmic reticulum calcium ATPase 2a (SERCA2a) (27). When RyRs channels are sensitized under non-physiological conditions, the higher levels of SR Ca^2+^ in atrial myocytes were more likely to spill over, resulting in spontaneous diastolic spark-mediated SR Ca^2+^ leakage (27–30). Notably, sparks with a certain scale and density may trigger the propagation of diastolic calcium waves. Propagating calcium waves can lead to NCX hyperfunction during diastole, producing DADs and triggering arrhythmias (27). On the other hand, the increased SR Ca^2+^ leakage during diastole is also accompanied by increased SR Ca^2+^ levels (owing to the increased SERCA2a activity). This combination of the leak and overload exacerbates propagated calcium waves and DADs and eventually results in atrial tachyarrhythmia (31, 32). A recent study demonstrated that ARNI can reverse the remodeling of RyR2 channels and NCX1 channels, which suggests that ARNI could correct the SR Ca^2+^ mishandling to a certain extent and contribute to reducing atrial arrhythmogenicity (33) (Figure 4).

An accumulating number of studies have shown that stress signaling pathways play a role in the electropathology of AF. It mainly involved the SR stress pathways, unfolded protein response (UPR) stress pathways, and mitogen-activated protein kinase (MAPK) stress signaling pathways. JUN N-terminal kinases (JNKs), as an important MAPK family member, are recognized as a marker of endoplasmic reticulum (ER) stress (34). In the Ca^2+^ handling process, on the one hand, JNK2 activates diastolic SR Ca^2+^ leakage, a known proarrhythmic effect, on the other hand, JNK2 accelerates the entry of Ca^2+^ into SR by stimulating SERCA2a activity, and the increased SR Ca^2+^ levels further aggravate diastolic SR Ca^2+^ leak, which together exacerbates the atrial arrhythmogenicity (31, 32, 35). Furthermore, JNK2-stimulated diastolic SR Ca^2+^ leakage is a key factor to activate calmodulin-dependent protein kinase II (CaMKII), which is a confirmed proarrhythmic signaling molecule (31, 35). In an experimental study, ARNI was validated to inhibit the p-Smad2/3, p-p38 MAPK, and p-JNK pathways, improve atrial tissue fibrosis, and reduce susceptibility to AF (36, 37) (Figure 4). In another rabbit study, ARNI was demonstrated to improve cardiac systolic function through the reduction of phosphorylated CaMKII expression and to avoid electrophysiological remodeling induced by MI (38).

Autonomic remodeling has a very significant impact on heart ion channels and may play an important role in the electropathology of AF (39). The increased sympathetic nerve densities were observed in atria from patients with AF and canine models (40, 41). Moreover, atrial sympathetic sprouting and neural hyper-innervation were observed in the cardiac ventricle after MI (42). The adrenergic effects that lead to arrhythmogenic triggers are highly related to Ca^2+^ handling. This effect intensifies almost all processes, such as Ca^2+^ entry, stores, and release in the heart, causing increased cardiomyocyte automaticity, EADs, or DADs-associated triggered activity, which strongly promotes the occurrence of arrhythmia (43–45). ARNI with the inhibition of neprilysin can result in an elevation in circulating NPs levels, which could enhance vagal tone and weakens sympathetic response, thereby exerting an antiarrhythmic effect (46).

Prior to this study, various studies have explored the effect of drugs on AF recurrence after ablation. One meta-analysis showed that the combined relative risk of AF recurrence in patients with RAAS inhibitors after ablation was 0.83 (95% CI: 0.70–0.98, *p* = 0.028), however, most studies that fulfill the inclusion criteria did not set a control group (47). Statins were found to reduce the recurrence of AF in the pooled randomized control trials, but not in all included studies (48). Colchicine was demonstrated to decrease the risk of AF recurrence within 3 months after ablation, however, the long-term efficacy of this drug remains to be further explored (49). Other non-cardiovascular drugs, such as steroids and omega-3 fatty acids for AF recurrence following RFCA have also been explored in other studies, but no significant benefit was found (50–52).

In addition to the effects of drugs, the development of new mapping tools may also help to improve the operation process and further improve the prognosis of patients with AF. In our study, the mapping catheters we used were LASSO electrodes, which have relatively few mapping points, low mapping density, cannot be automatically mapped, and usually required manual correction. In recent years, many high-density and ultra-high-density mapping electrodes, such as PentaRay catheters, Orion catheters, and HD Grid catheters have been developed, and some are currently used in clinical applications (53, 54). The Orion basket electrodes had high electrode density, with a total of 64 electrodes located in 8 basket branches. However, it is costly, technically difficult, and incompatible with a contact force sensing catheter. The HD Grid electrodes can record 32 bipolar signals at the same time, which can more effectively and accurately identify the voltage area, but it has not yet been applied to clinical practice in China. PentaRay electrodes can achieve safe and precise mapping with less manual correction, which is significantly better than LASSO electrodes. These high-density catheters can not only be beneficial in optimizing individualized treatment but also in achieving zero X-ray ablation without affecting the safety and effectiveness of the ablation. At present, the concept of zero X-ray ablation was becoming more important in the practice of interventional cardiology (55, 56). Since radiation exposure could lead to major adverse impacts, such as cognitive impairment, long-lasting or permanent infertility, and malignancies in patients and interventional workers, and the long-term use of lead apron was also strongly associated with neck and back pain and a high incidence of multilevel disk disease (55).

This study had several limitations. First, anatomical variations of the PV are related to the operation strategy and the long-term effects of ablation. Left common PV (LCPV) is a very common type of variation, and some patients may manifest a short common PV. Since the two left PVs are very close, selective pulmonary vein angiography is often difficult to detect these variations, whereas cardiac CT can show these small anatomical structures. LCPV may not only facilitate greater contact force along the left PV-left atrial appendage ridge but also deeper ablation, and thus may help to improve patient outcomes. In addition, LCPVs may more frequently harbor triggers responsible for AF episodes. Other variations, such as accessory PVs may also influence the outcome of ablation, but further research is still needed. During our procedure, a wide area circumferential antral ablation technique was used to perform PVI. Although popular, the incidence of long-term PV reconnection of this technique remains high, and a study is trying to improve this technique (57). After PVI, an AF induction test was repeated, and only reproducible non-PV triggers were mapped. When AF was induced and a non-PV trigger was suspected, mapping catheters were re-positioned in the atria, such as the SVC, LA posterior free wall, or atrial septum, according to the possible region of non-PV triggers estimated from the intracardiac activation pattern. However, additional ablation still needs more supporting clinical evidence. Second, the proportion of patients reaching the target dose was lower in both groups, which may have resulted in the clinical benefit of both ACEI and ARNI not being adequately exhibited. Nonetheless, the data represent a real-world clinical setting. Third, the recurrence rate of AF may be underestimated due to some patients with AF recurrence being asymptomatic, and our follow-up protocol did not include long-term ECG monitoring. Finally, our study was a retrospective clinical study, so it can only be considered a preliminary study and needs to be verified in future RCTs.

## Conclusion

In this study, we found that, compared with ACEI, ARNI was associated with a lower risk of AF recurrence in patients who underwent RFCA for AF. Large-scale RCTs are needed to verify these pioneer findings.

## Data availability statement

The raw data supporting the conclusions of this article will be made available by the authors, without undue reservation.

## Ethics statement

The studies involving human participants were reviewed and approved by the Ethics Committee of The Second Affiliated Hospital of Nanchang University. Written informed consent for participation was not required for this study in accordance with the national legislation and the institutional requirements.

## Author contributions

YD and JL designed the study. KS, SC, and DL collected the data. SX, JH, and ZZ analyzed the data. YD, BH, JL, and ZZ wrote this manuscript. All authors reviewed the manuscript.

## Footnotes

1
http://www.empowerstats.com
